# Potential Role of Thymosin-*α*1 Adjuvant Therapy for Glioblastoma

**DOI:** 10.1155/2009/302084

**Published:** 2010-01-11

**Authors:** Arno Sungarian, Deus Cielo, Prakash Sampath, Nathaniel Bowling, Peter Moskal, Jack R. Wands, Suzanne M. de la Monte

**Affiliations:** ^1^Departments of Medicine, Pathology, and Neurosurgery, Rhode Island Hospital and the Warren Alpert Medical School, Brown University, Providence, RI 02903, USA; ^2^Pierre Galletti Research Building, Rhode Island Hospital, 55 Claverick Street, Room 419, Providence, RI 02903, USA

## Abstract

Glioblastomas are high-grade, malignant CNS neoplasms that are nearly always fatal within 12 months of diagnosis. Immunotherapy using proinflammatory cytokines such as IL-2 or IL-12 may prolong survival with glioblastoma. Thymosin-*α*1 (Talpha1) is a thymic hormone and immunemodulator that increase IL-2 production and T-cell proliferation. We examined potential therapeutic effects of Talpha1 in experimental in vivo glioblastoma, and characterized Talpha1's anti-tumor effects in vitro. Rar 9L cells (10^4^) were implanted into the right frontal lobe of adult Long Evans rats that were subsequently treated with vehicle, BCNU, Talpha1, or Talpha1+BCNU from postoperative day 6. Talpha1+BCNU significantly lowered tumor burdens, and increased cure rates. In vitro experiments demonstrated that Talpha1 had no direct effect on viability or mitochondrial function, and instead, it increased expression of pro-apoptosis genes, including FasL, FasR and TNF*α*-R1 (65.89%, 44.08%, and 22.18%, resp.), and increased 9L cell sensitivity to oxidative stress. Moreover, Talpha1 enhanced 9L cell sensitivity to both Granzyme B- and BCNU-mediated killing. The findings suggest that Talpha1 enhances BCNUmediated eradication of glioblastoma in vivo, and that Talpha1 mediates its effects by activating pro-apoptosis mechanisms, rendering neoplastic cells more sensitive to oxidative stress and immune-mediated killing by Granzyme B and chemotherapeutic agents.

## 1. Introduction

Glioblastoma is the most common primary CNS malignant neoplasm in adults, accounting for nearly 75% of the cases. Despite steady progress in treatment due to improvements in neuro-imaging, microsurgery, and radiation, glioblastomas remain incurable [1–5]. The mean survival rate is less than one year from diagnosis, and the five-year survival rate following aggressive therapy including gross tumor resection is less than 10% [1, 6–8]. Glioblastomas cause death due to rapid, aggressive, and infiltrative growth, which renders the tumors unresectable in toto. Additional problems compounding treatment and cure are that glioblastomas are relatively resistant to radiation and chemotherapy [1, 2, 6–9], and the host immune response to the tumors is likely to be ineffective in eradicating even small populations of neoplastic cells that remain following conventional treatment including surgery [10–12]. 

Malignant glioma cells can evade detection by the host's immune system by producing immunosuppressive peptides that impair T-cell functions such as proliferation and production of IL-2 [10]. The CNS is also somewhat immune privileged, which contributes to the unfettered growth of malignant neoplastic cells. Immunotherapy or treatment by recruitment of the immune system to kill malignant neoplastic cells has been researched in many models. Thymosin fraction 5 (TF5), thymosin-*α*1 (Talpha1/thymalfasin), IFN-*α*, and IL-2 are among the many immune-related components that have been studied for their abilities to fight malignant neoplasms. Talpha1 is a 28-amino acid peptide, and a synthetic form of a naturally occurring hormone that circulates in the thymus [13–15]. Talpha1 and thymic peptide hormones stimulate thymocyte growth and differentiation, production of IL-2, T cell IL-2 receptors, IFN-*γ* and IFN-*α* [16–26]. Talpha1 has been used in clinical trials to treat Hepatitis B and Hepatitis C virus infections [13, 27–34], malignant neoplasms [14, 15, 35], cytomegalovirus infection following renal transplant [36], and AIDS [13]. The promising results of these investigations, combined with the evidence of reduced T-cell responsiveness to glioblastomas, led to the present work evaluating the potential therapeutic benefit of Talpha1 immunotherapy for treating malignant gliomas, and determining the mechanisms in which Talpha1 exerts its antineoplastic effects.

## 2. Experimental Design

### 2.1. In Vivo Studies

Experiments were designed to determine if Talpha1 delivered alone or in combination with Bischloroethylnitrosourea (BCNU) could significantly reduce glioblastoma burden compared with BCNU treatment alone. Experiments were conducted using an in vivo model of glioblastoma generated in adult (250–300 grams) Long Evans rats. Rats were anesthetized with a single intraperitoneal injection of 100 mg/kg xylazine and 15 mg/kg ketamine in 14.2% ethanol. Under deep anesthesia, the heads were shaved and prepped with povidone iodine and the rats were securely positioned in a stereotaxic frame. A midline incision was made and a 3 mm burr hole was drilled over the right frontal lobe. A Hamilton syringe equipped with a 26-gauge needle was fixed at the center of the burr hole. The needle was placed at a depth of 3.5 mm for injection of 1 × 10^4^ cells in a volume of 2 *μ*L. After removing the needle, the wound was washed with sterile saline and the skin was closed with self-resorbing sutures. The rats were returned to their cages and observed for any signs of deterioration including weight loss, reduced food and water intake, hemiplegia, seizures, or inactivity. Empiric studies demonstrated that intracerebral injection of 10,000 9L cells kills 100% of the rats within 21–24 days of implantation. These studies were approved by the Lifespan/Rhode Island Hospital IACUC Animal Care and Use Committee. It should be noted that the Lifespan/Rhode Island Hospital IACUC Animal Care and Use Committee guidelines do not permit animals to die from experimental disease. Therefore, survival studies could not be performed, and instead, the rats were sacrificed at predetermined intervals to assess tumor burden.

Rat 9L glioblastoma cells were obtained from the American Type Culture Collection (Washington, D.C.) and certified as pathogen-free. The cells were maintained in Dulbecco's Modified Eagle's Medium (DMEM) supplemented with 5% heat-inactivated fetal calf serum (FCS), 2 mM glutamine, and 100 *μ*M nonessential amino acids (Gibco-BRL, Grand Island, NY). Antibiotics were not added to the medium. Prior to injection, the cells were rinsed with phosphate-buffered saline (PBS), detached from the culture surfaces and dissociated into single cell suspensions with 0.25% trypsin/0.05% EDTA. The dissociated cells were washed 3 times in serum-free DMEM and finally suspended in serum-free DMEM at a density of 5 × 10^6^ viable cells/mL. Viable cell density was determined using Trypan blue exclusion and a hemocytometer chamber. 

Rats were divided into 6 treatment groups and all treatments were either given or initiated on day 6 following intracerebral 9L cell inoculation. The groups were as follows: vehicle control (*N* = 17); low dose (45 *μ*g/kg; *N* = 19) Talpha1; high dose (200 *μ*g/kg; *N* = 20) Talpha1; low dose Talpha1+BCNU (*N* = 18); high dose Talpha1+BCNU (*N* = 24); BCNU only (*N* = 26). BCNU (4.4 mg/kg) was administered as a single intraperitoneal (i.p.) injection. The indicated doses of Talpha1 were given on 3 consecutive days by i.p. injection. Human recombinant Talpha1 was used in these experiments. Previous studies demonstrated significant immuno-modulating effects of human Talpha1 in rat cells, both in vivo and in vitro [37]. Two different termination points were used in the experiments, day 17 and day 21 after the 9L cell inoculation. The rats were sacrificed by i.p. injection of 120 mg/kg sodium pentobarbital. The brains were harvested, sectioned, immersion fixed in Histochoice (Amresco Co., Solon, Ohio), and processed to generate hematoxylin and eosin stained paraffin embedded sections to evaluate the histopathology.

### 2.2. In Vitro Studies

#### 2.2.1. Cell Lines and Talpha1 Treatments

To evaluate the effects Talpha1 on viability, mitochondrial function, and protein expression, 9L cells were seeded into 96-well plates at a density of 2 × 10^4^ cells per well. In order to demonstrate mechanisms of Talpha1 adjuvant cell killing, we used established tumor cell lines instead of primary brain tumors because consistency across all assays and within each experiment was needed. In addition, 9L cells were used for the in vivo experiments, and it was important to understand how Talpha1 treatment facilitates inhibition of tumor growth in the brain. After overnight attachment and growth, the cells were exposed to 10^−5^ M Talpha1 for 24 hours, and then evaluated for viability or gene expression. To study chronic effects of Talpha1 treatment, cells seeded in 75-mm^2^ flasks were exposed to 3 consecutive daily treatments of 10^−5^ M Talpha1 (added to fresh medium), after which the cells were trypsinized and reseeded into 96-well plates at a density of 2 × 10^4^ viable cells per well. After 24 hours of further Talpha1 treatment, the cells were evaluated for viability or gene expression. The optimum concentration of Talpha1 was determined from initial dose-toxicity studies in which 9L cells and postmitotic primary rat cortical neuron cultures were treated with different concentrations of Talpha1 ranging from 10 nM to 50 *μ*M. Primary cortical neuron cultures were studied to enable selection of Talpha1 doses that would not be toxic to nonneoplastic brain cells. Cell viability and mitochondrial function were measured using the Crystal violet (CV) and MTT assays, respectively, since previous studies showed that CV and MTT absorbances increase linearly with cell density from 1 × 10^4^ to 5 × 10^5^ cells per well [38–40]. The studies were extended to determine if Talpha1 treatment increased cellular sensitivity to oxidative stress or BCNU by exposing the Talpha1-treated cells to H_2_O_2_ (100 *μ*M) or BCNU (25 *μ*M) for 24 hours, and evaluating viability and mitochondrial function using the CV and MTT assays.

#### 2.2.2. Directional Motility Assay

Directional motility was measured using Boyden chamber-type culture inserts that had 8 *μ*M pore membranes [41]. The chambers were seated in 24-well plates. 10^5^ viable cells were placed into the upper chamber in serum-free medium. Medium supplemented with 1% FCS was placed in the well below to provide a trophic stimulus for migration. Migration was allowed to proceed for 4 hours. ATPLite (Packard Instrument Company, Meriden, CT) was used to quantify viable cell density on the upper surface of the membrane (nonmotile), the undersurface of the membrane (migrated adherent), and at the bottom of the well (migrated, nonadherent). Briefly, nonmotile cells were removed from the upper surface of the membrane using a cotton swab. The cells were lysed by immediately submerging the swabs into 200 *μ*L of diluted ATP lysis solution in a well of a black 96-well microplate. Completeness of cell harvesting was monitored microscopically. Cells adherent to the undersurface of the membrane were harvested and lysed by submerging the wiped membrane in 200 *μ*L of diluted ATP lysis solution in a second well of a black microplate. Cells in the well below the culture insert were resuspended and added directly to 25 *μ*L of undiluted ATP lysis solution in a third well of a black microplate. After 5-minute incubation with agitation to ensure complete cell lysis, ATPLite substrate (25 *μ*L) was added to each well. The reactions were mixed for 2 minutes by gentle platform agitation. Subsequently, the plates were dark adapted for 5 minutes and then luminescence was measured in a TopCount Microplate reader (Packard Instrument Company, Meriden, CT). The percentages of motile adherent, motile nonadherent, total population of motile cells were calculated per chamber [41]. For each experiment, replicates of 4–6 assays were performed, and the results were analyzed statistically. In preliminary studies, we demonstrated that ATPLite luminescence increases linearly with cell number between 10^3^ and 5 × 10^5^ cells, as indicated by the manufacturer.

#### 2.2.3. Microtiter Immunocytochemical ELISA (MICE) Assay

The MICE assay is a rapid and sensitive method of quantifying immunoreactivity in 96-well microcultures and combines the advantages of the enzyme-linked immunosorbent assay with immunocytochemical staining to permit sensitive in situ quantification of protein expression with values normalized to cell density [38]. Briefly, the fixed cells were permeabilized with 0.05% saponin in Tris-buffered saline (50 mM Tris, pH 7.5, 0.9% NaCl; TBS), blocked with Superblock-TBS (Pierce, Rockford, IL), and then incubated overnight at 4°C with primary antibodies to proliferating cell nuclear antigen (PCNA), bcl-2, p21/Wwaf-1, p53, FasL, FasR, TNF-R1, or GAPDH, each diluted to 0.5–1 mg/mL in TBS containing 0.05% Tween-20 and 0.5% bovine serum albumin (TBST-BSA). Immunoreactivity was detected using horseradish peroxidase conjugated secondary antibody (Pierce, Rockford, IL) and the TMB soluble substrate (Pierce, Rockford, IL). Reactions were stopped by the addition of 1 M H_2_SO_4_ and absorbances were measured at 450 nm in a Spectracount microplate reader (Packard Instrument Co., Meriden, CT). Subsequently, cell density was measured by staining the adherent cells with Coomassie blue dye and measuring the absorbance of the eluted dye [38]. The MICE index was the calculated ratio of TMB and Coomassie blue absorbance measured in the same culture well. Mean ± S.D. of results obtained from 16 replicate culture wells was used for intergroup statistical comparisons.

#### 2.2.4. Granzyme-B Experiments

T cells and natural killer (NK) cells utilize two major cytotoxic pathways including perforin-granzyme-mediated necrosis and tumor necrosis factor (TNF) family ligand-mediated apoptosis [42–48]. To determine if the antineoplastic effects of Talpha1 were mediated through perforin/Granzyme B-induced killing, we employed a novel in vitro assay that quantified Granzyme B-mediated killing with no requirement to add well-characterized and activated cytotoxic T cells and NK cells. To accomplish this, 9L cells were first treated with Talpha1 or vehicle for 24 hours, after which they were harvested, reseeded into 96-well black plates (7.5 × 10^5^ cells/75 *μ*L/well), and exposed to 20,000 units/mL Streptolysin O (SLO) plus 100 ng recombinant Granzyme B (reaction volume 100 *μ*L) for 1 or 3 hours at 37°C. The SLO was used in place of perforin to permeabilize the cells [49], and recombinant Granzyme B was used to standardize the assay. Control studies included parallel reactions in which SLO, Granzyme B, or both were omitted. Viable cell density was measured using the ATPlite assay (Packard Instrument Company, Meriden, CT), which has a broad linear dynamic range correlating relative light units with cell densities between 10^3^ to 10^6^ cells per culture well.

#### 2.2.5. Source of Reagents

Recombinant human Talpha1 was provided as a gift from Sciclone, Inc. (San Francisco, CA). Monoclonal antibodies to PCNA, p53, bcl-2, FasR, FasL, TNF-R1, and p21 were purchased from Santa Cruz biotechnology, Inc., (Santa Cruz, CA), and monoclonal antibodies to glyceraldehyde-3-phosphate dehydrogenase (GAPDH) were purchased from Chemicon International, Inc. (Temecula, CA). Granzyme B was obtained from CalBiochem (Carlsbad, CA). Streptolysin O was purchased from Sigma-Aldrich, St. Louis, MO.

#### 2.2.6. Statistical Analysis

Data depicted in the graphs represent the mean ± S.D. of results. Intergroup comparisons were made with the Student's *t*-test or analysis of variance and the Fisher Least Significance post hoc significance test using the Number Cruncher Statistical Systems (Dr. Jerry L. Hintze, Kaysville, UT).

## 3. Results

### 3.1. Glioblastoma Model

Intracerebral inoculation of adult Long Evans rats with 10,000 9L rat glioblastoma cells consistently produced tumors that progressively enlarged and caused death within 21–24 days. In untreated rats, the time-dependent progression of tumor growth and associated clinical signs were characterized as follows: (1) increased physical activity with tumor diameters of 4-5 mm at day 7; (2) hyperresponsiveness by day 14 with tumor diameters of 7-8 mm and frequent associated intratumor hemorrhage; (3) somnolence with tumor masses of 10–12 mm accompanied by cerebral herniation by day 21-22. Histological sections stained with hematoxylin and eosin confirmed the presence of large tumor masses composed of infiltrative malignant neoplastic cells. Histopathological studies of coronal sections through the entire cerebrum demonstrated that within the first 24 hours of inoculation, no grossly visible tumor could be detected. However, after 5 days, the 9L cells had grown and formed small tumor masses that were localized in the superficial cortex and overlying leptomeninges. Within 10 days, the tumor masses extended to deeper structures including the basal ganglia and the walls of the lateral ventricles and were associated with moderate edema but no herniation (shift of midline structures). By day 14, the tumor masses were found to extend deeply within the cerebral hemispheres and occupy nearly 50% of the cross-sectional area of the section with associated edema and early herniation. Despite the large size, the demarcation between tumor mass and cerebral parenchyma remained sharp. By day 21 or 22, the tumor masses occupied nearly the entire right frontal lobe with variable degrees of extension to the contra-lateral hemisphere. The extensive tumor mass was associated with marked cerebral edema, hemorrhage, and herniation.

### 3.2. Effects of Talpha1 and BCNU on Glioblastoma Growth (Figure 1)

A semiquantitative histological grading scale was used to assess tumor burden for intergroup comparisons: 0: no residual tumor; 1: microscopic tumor confined to the superficial cortex; 2: tumor mass occupying less than 25% of the hemisphere cross section; 3: tumor mass occupying up to 50% of the hemispheric cross-section and extending into deep structures; 4: massive tumor burden with involvement of 50–90% of the ipsilateral hemispheric cross-section with extension of tumor across the midline into the contralateral hemisphere. The sections were coded and graded simultaneously by two of the investigators (SMD and AS) without knowledge of treatment group. To verify consistency in the grading, all samples were shuffled and rereviewed under code.

**Figure 1:**
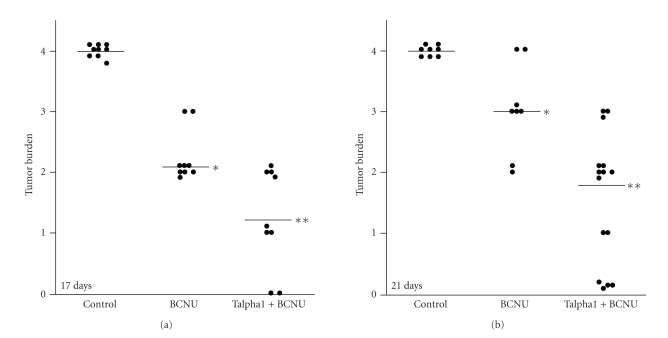
Analysis of tumor burden in rats treated with vehicle (control), BCNU (4.4 mg/kg), or BCNU (4.4 mg/kg)+Talpha1 (45 mg/kg). (a) depicts results obtained 17 days after tumor cell implantation. (b) depicts results obtained 21 days after tumor cell implantation. Each dot represents an individual rat case. Closed circles on the 0 line indicate cures. The horizontal bars correspond to the group mean score. Intergroup statistical comparisons demonstrated significantly lower tumor burdens in BCNU-treated relative to control (*P < .01), and between Talpha1 and control (*P* < .001**) or BCNU-treated rats (*P* < .01**).

Since spontaneous tumor rejections were not observed in preliminary studies, all experiments were terminated on days 17 or 21 after 9L cell implantation. Representative data from one of the experiments (replicated 3 times) are shown in Figure 3. Using the standardized grading scheme to assess tumor burden, we demonstrated that 100% of vehicle-treated rats had Grade 4 tumor burdens by day 17 (Figure 1), similar to the effects of no treatment (data not shown). In contrast, rats treated with BCNU exhibited significant reductions in tumor mass relative to vehicle-treated controls (*P* < .01). On Day 17, the tumor burden was Grade 2 in 75%, and Grade 3 in 25% of those treated with BCNU only; whereas on Day 21, 25% of the group each had Grades 4 or 2 tumor burden, and 50% had Grade 3 tumor burden. In essence, the effectiveness of BCNU was nonuniform with nearly half the group manifesting no apparent therapeutic response, while the other half had their expected tumor burdens reduced by up to 50% relative to control. Rats treated only with Talpha1 had tumor growth and clinical deterioration rates that were similar to control (data not shown). In addition, rats treated only with either the 45 *μ*g/kg and 200 *μ*g/kg doses of Talpha1, the clinical course was complicated by extreme intracerebral swelling and substantially greater degrees of herniation (brain tissue protruding through the Burr hole) compared with all other groups. Similarly, in rats treated with BCNU+200 *μ*g/kg of Talpha1, marked degrees of cerebral swelling caused increased morbidity and mortality. Therefore, studies using Talpha1 alone or 200 *μ*g/kg Talpha1+BCNU were discontinued. Remarkably, the 45 *μ*g/kg Talpha1+BCNU group had significantly lower mean tumor burdens relative to vehicle-treated and BCNU-treated rats, at both the 17 and 21 days time points (*P* < .001; see Figure 1). On Day 17, 44.4% of those cases exhibited Grade 2 tumor burden, 33.3% Grade 1, and 22.2% were cured. On Day 21, 20% had Grade 3 tumor burdens, 40% had Grade 2 tumor burdens, 13.3% had Grade 1 tumor burdens, and 26% were cured. Cure rates among rats treated with Talpha1+BCNU were significantly higher than all other groups (*P* < .001).

Histopathological studies were used to evaluate the effects of BCNU and Talpha1+BCNU in relation to tumor growth in vivo. In vehicle-treated control rats, the tumors grew as densely cellular neoplasms with foci of spontaneous necrosis, frequent mitoses, prominent nuclear pleomorphism (variability in cell size and shape), and irregularly infiltrating borders (data not shown). In BCNU-treated rats in which tumor growth was reduced, the histopathological studies revealed larger foci of tumor necrosis with accompanying mononuclear inflammatory cell infiltrates composed of macrophages and lymphocytes. In rats treated with 45 mg/kg Talpha1+BCNU, the marked reductions in tumor burden were associated with large areas of cavitary necrosis and accompanying mononuclear inflammatory cell infiltrates composed of macrophages and lymphocytes. In cases where no residual tumor was found, only small scars or cavities marked the location of the previous tumors.

### 3.3. Effects of Talpha1 on 9L Cell Viability

**Figure 2:**
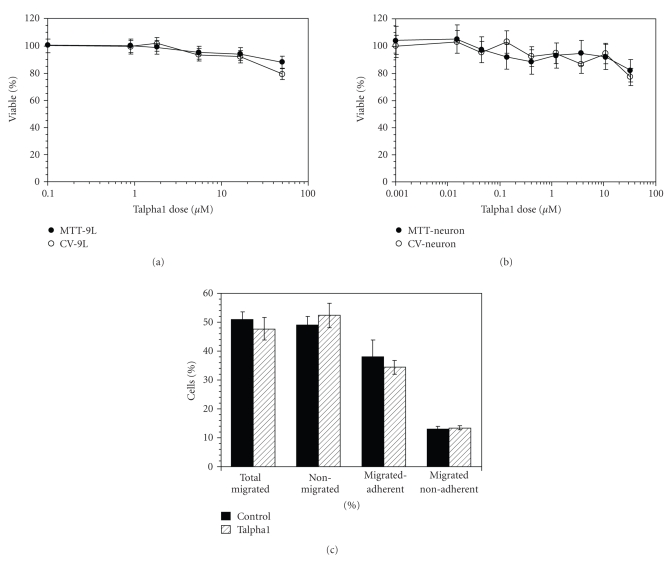
Talpha1 is not directly cytotoxic for (a) rat 9L glioblastoma cells or (b) normal rat cortical neurons, and (c) does not inhibit cell migration. 9L cells and cortical neurons were cultured in 96-well plates and treated with different concentrations of Talpha1. Viability was measured using the Crystal violet assay, and mitochondrial function was measured using the MTT assay. Both the 9L cells and cortical neurons remained viable and exhibited minimal reductions in mitochondrial function over a broad range of Tapha1 exposure. In cultures treated with 50 mM Talpha1 (highest dose used), both cell types exhibited approximately 20% reductions in MTT and CV indices; however, that concentration was considerably higher than the amounts used for the in vivo experiments. (c) In vitro motility assays were performed using 9L cells that were treated with vehicle or Talpha1 (10 mM) for 24 hours. Motility was measured using the ATP Luminescence-Based Motility/Invasion assay (see Methods). The percentages of total migrated, nonmigrated, migrated-adherent (passed through the pores and remained adherent to the undersurface of the membrane), and migrated nonadherent cells (passed through the pores and landed at the bottom of the chamber) were calculated, and the means ± S.D.s of the results are depicted graphically.

In vitro experiments were conducted to determine if the in vivo effects of Talpha1 on 9L glioblastoma cell growth were mediated by direct cytotoxic actions or indirect mechanisms. To perform these studies, 9L cells were seeded in 96-well plates and exposed to Talpha1 for 24 hours, after which the cultures were analyzed for viability using the Crystal violet assay, and mitochondrial function using the MTT assay. Mitochondrial function was measured because cell death can be mediated by mitochondrial dysfunction rather than apoptosis or necrosis. Although slight dose-dependent reductions in viability and MTT activity were observed, the overall cytotoxic effects of the Talpha1 were relatively modest (Figure 2(a)). Similar results were obtained with respect to primary cortical neuron cultures (Figure 2(b)), *as well as other cell lines, including 293 kidney cells and SH-Sy5y neuroblastoma cells (data not shown).* It is noteworthy that the highest concentration of Talpha1 used in the in vitro experiments (50 mM), which produced 20% declines in the mean levels of viability and mitochondrial function, was nearly 1000-fold higher than the doses used for in vivo experiments in which tumor burdens were observed to be substantially reduced. These results indicate that Talpha1 does not have direct cytotoxic effects on 9L glioblastoma cells, and that other factors contributed to the adjuvant therapeutic effects observed in vivo.

### 3.4. Studies of Talpha1 Effects on 9L Cell Motility

Directional motility assays were used to determine if the growth inhibitory effects of Talpha1 were mediated by reduced cell motility and invasiveness. The ATP-Luminescence-based motility assay enables quantification of the total percentages of migrated cells, as well as the percentages of the migrated adherent and migrated nonadherent subpopulations. Treatment with Talpha1 did not significantly alter 9L cell motility or adhesiveness (percentage of migrated-adherent cells) (Figure 2(c)). Similarly, in further studies using human 293 kidney or human SH-Sy5y neuroblastoma cells, Talpha1 treatment was found to have no significant effect on directional motility or cell adhesion (data not shown). Therefore, it is unlikely that the reduced tumor mass in Talpha1-treated rats was due to inhibition of tumor cell migration, adhesion, or invasive growth.

### 3.5. Effects of Talpha1 on Proapoptosis and Prosurvival Gene Expression

**Figure 3:**
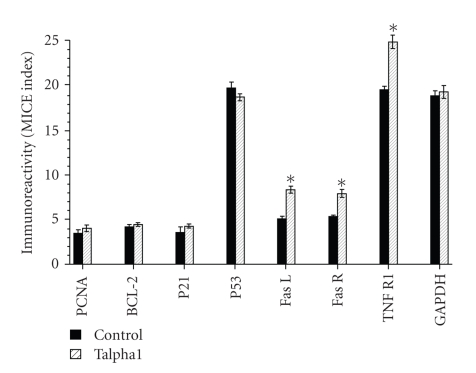
Increased proapoptosis gene products in Talpha1-treated 9L glioblastoma cells. 9L cells seeded in 96-well plates were treated with 10 mM Talpha1 for 24 hours. After fixation and permeabilization, immunoreactivity was measured directly in the cells using the microtiter immunocytochemical ELISA (MICE) assay. Levels of immunoreactivity were normalized to cell density and the corresponding ratio represents the MICE index. The graph depicts the mean ± S.D. of the levels of proliferating cell nuclear antigen (PCNA), Bcl-2, p21, p53, Fas L, Fas R, tumor necrosis factor receptor, type 1 (TNF R1), and glyceraldehyde-3-phosphate dehydrogenase (GAPDH) measured in 8 replicate vehicle-treated or Talpha1-treated cultures. Asterisks denote significant differences from control (*P* < .01).

Since Talpha1 did not have direct cytotoxic effects, we explored other potential mechanisms by which Talpha1 could function to inhibit growth of glioblastomas. In this regard, we examined the expression of gene products that promote either apoptosis or cell survival, and assessed housekeeping gene expression as control. The studies were performed in 96-well plates using the microtiter immunocytochemical ELISA (MICE) assay to generate data from multiple replicate cultures. Talpha1 treatment for 24 hours resulted in significantly increased levels of FasR (44.1%), FasL (65.9%), and TNF-R1 (22.2%) relative to vehicle-treated controls (*P* < .01; see Figure 3). In contrast, expression levels of bcl-2, p21/waf1, p53, proliferating cell nuclear antigen (PCNA), and glyceraldehy-3-phosphate dehydrogenase (GAPDH) were not significantly affected by the Talpha1 treatment. Similar studies performed with human 293 kidney or SH-Sy5y neuroblastoma cells demonstrated significant reductions in bcl-2 (42% and 36.7%, resp.; *P* < .01), but no significant changes in the levels of FasR, FasL, or TNF-R1 expression (data not shown).

### 3.6. Talpha1 Increases 9L Cell Sensitivity to Oxidative Stress and BCNU Treatment

**Figure 4:**
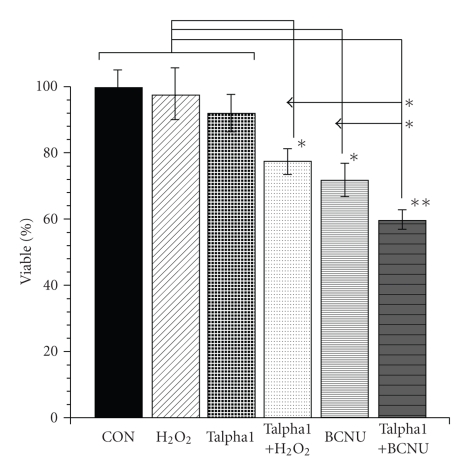
Talpha1 increased 9L cell sensitivity to oxidative stress and BCNU killing. 9L cells were seeded in 96-well plates. After overnight attachment and growth, cells were pretreated with 10 mM Talpha1 (Ta1) or vehicle. 12 hours later, the medium was changed and cells were treated with vehicle (control), 0.1 mM H_2_O_2_, Talpha1 only (Ta1), Talpha1+H_2_O_2_, BCNU (25 mM), or Talpha1+BCNU. Viability was measured in 12 replicate cultures using the crystal violet assay. The graph depicts the mean ± S.D. of results. Asterisks indicate significant differences (*P* < .01) from vehicle-treated control, H_2_O_2_-treated, and Talpha1-only treated cultures.

Activation of proapoptosis mechanisms or inhibition of survival pathways could increase cellular sensitivity to oxidative stress and cytotoxic agents. Therefore, studies were conducted to determine if Talpha1-treated cells were rendered more sensitive to oxidative stress or killing by BCNU. Cells treated with 100 mM H_2_O_2_ or 10 mM Talpha1 exhibited similar mean levels of viability relative to vehicle-treated control cells (Figure 4). Treatment with BCNU alone resulted in 28% cell loss relative to control (*P* < .01). Pretreatment with Talpha1 for 24 hours resulted in significantly reduced viability in cultures that were subsequently exposed to a sublethal dose (100 mM) of H_2_O_2_  (*P* < .01), and significantly greater cell loss in cultures treated with Talpha1+BCNU compared with cultures that were treated with BCNU only (*P* < .01), or H_2_O_2_, Talpha1, or vehicle (*P* < .001) (Figure 4). Similar results were obtained using 293 or C6 glioma cells as targets in the assay (data not shown).

### 3.7. Talpha1 Increases 9L Cell Sensitivity to Granzyme B-Mediated Killing

**Figure 5:**
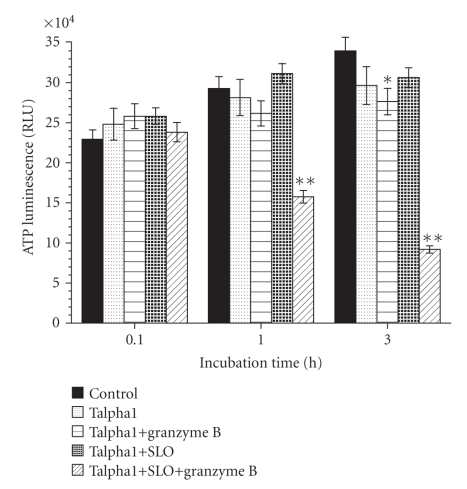
Talpha1 enhances Granzyme B-mediated killing of 9L glioblastoma cells. 9L cells were pretreated with vehicle or Talpha1 for 3 hours. The cells were then divided and further treated with vehicle, Streptolysin O (permeabilizing agent to substitute for Perforin), Granzyme B only, or Granzyme B+Streptolysin O. After 0.1, 1, or 3 hours of incubation, the cells were harvested to determine viability (resistance to killing). Each experiment was performed in replicates of 4, and cell viability, measured with the ATP luminescence, was assayed in replicates of 6 or 8 *P < .001 and *P < .05 relative to control.

In vivo malignant neoplastic cells are killed by cytotoxic T cells and macrophages that are recruited by proinflammatory cytokines such as IL-2 and IL-12. Cytotoxic T cells kill by releasing perforin, which generates holes in target cell membranes, and Granzyme B, which causes enzymatic destruction and death of the target cells. To study the potential role of Talpha1 in enhancing T cell-mediated killing of 9L glioblastoma cells, we constructed an in vitro assay in which Talpha1- or vehicle-treated 9L cells were incubated with Granzyme B in the presence or absence of Streptolysin O (permeabilizing agent) for 1 or 3 hours. Viability was measured using the ATP luminescence assay and within group comparisons were made to determine the relative killing associated with SLO+Granzyme B treatment. The studies demonstrated significant reductions in cell viability in Talpha1-treated cultures that were exposed to SLO+Granzyme B relative to Talpha1-treated cultures exposed to SLO, Granzyme B, or vehicle only (*P* < .001; Figure 5). In addition, Granzyme B-mediated killing progressed over time as evidenced by the substantially greater degrees of cell loss observed in assays performed after 3 hours compared to 0.1- or 1-hour incubation (Figure 5). In addition, after 3 hours of incubation, cultures treated with Talpha1+Granzyme B also had reduced viability relative to control (*P* < .05), suggesting modest slow (inefficient) cellular entry of Granzyme B in the absence of SLO.

## 4. Discussion

Glioblastomas are highly aggressive primary CNS malignant neoplasms that kill by widespread infiltration of the brain parenchyma and mass effect. Despite a number of advances in understanding the molecular genetics of these neoplasms and in devising novel treatments, a major barrier for achieving effective disease control or cure is the generally lackluster host immune response to the neoplastic cells. Due to their infiltrative growth patterns, it will probably never be possible to achieve total curative surgical excision of glioblastomas, and therefore other approaches must be utilized. However, even with the application of high-dose chemotherapy or radiation, the host response is critical for removing debris and eradicating small populations of residual and newly arising neoplastic cells. With glioblastomas, suboptimal function of this effector arm may account for the repeated and rapid recurrences of tumor following apparently successful treatment. In this regard, recent evidence indicates that immune modulator therapy may prolong survival and promote glioblastoma killing in vivo. 

Talpha1 (Thymosin-*α*1) is a 28-amino acid peptide representing a synthetic form of a naturally occurring compound found in circulation [14, 15]. Talpha1 stimulates thymocyte growth and differentiation, production of IL-2, T-cell IL-2 receptors, IFN-*γ* and IFN-*α* [16–24, 26, 50]. Talpha1 has been used in clinical trials to treat hepatitis B and C infections [13, 27–33], carcinomas of the head and neck, lung cancer, and malignant melanoma [14, 15, 35], cytomegalovirus infection following renal transplant [36], and AIDS [13]. The promising results of these clinical investigations, combined with the evidence of reduced T-cell responsiveness to glioblastomas, led to the present work evaluating the potential therapeutic benefit of Talpha1 immunotherapy for treating malignant gliomas and determining the mechanisms in which Talpha1 exerts its antineoplastic effects. Further interest in the potential adjuvant use of Talpha1 for treating malignant gliomas stems from the facts that (1) it represents a synthetic purified version of a endogenously produced compound and thus minimal evidence can be found of clinical toxicity in humans; (2) previous independent work demonstrated that Talpha1 stimulates production of proinflammatory cytokines [17–22, 26, 50–55], which provide a therapeutic benefit in slowing the progression of experimental malignant glial neoplasms.

The in vivo studies using an experimental model of glioblastoma demonstrated that while BCNU treatment did significantly reduce tumor burden, the responses were heterogeneous with several cases exhibiting no detectable effect relative to vehicle-treated controls. However, treatment with Talpha1+BCNU provided significant therapeutic benefit both with respect to reducing mean tumor burden and curing tumors in approximately 25% of the cases. The Talpha1+BCNU-mediated reductions in tumor burden were associated with increased tumor cell necrosis accompanied by lympho-mononuclear cell infiltrates within and around the residual mass of neoplastic cells in the brain. In cases where no residual tumor could be found, only gliotic scars or cavitated foci with associated scant inflammation were detected. Glioblastoma cures were observed in approximately 25% of the low-dose (45 mg/kg) Talpha1+BCNU treated group. 

A somewhat unexpected finding was that the Talpha1-only groups, and rats treated with 200 mg/kg Talpha1+BCNU fared the same as control or worse. Frequently, the brains of Talpha1-only treated rats were more swollen and herniated due to inflammation and edema combined with the growing tumor mass. In this regard, it is noteworthy that rats treated with the lower concentration of Talpha1 ± BCNU had better outcomes than those treated with the higher concentration of Talpha1 ± BCNU since the tumor cell killing was similar, but edema and herniation were more prevalent and pronounced in the group that received the higher concentration of Talpha1. Therefore, Talpha1 treatment alone did not eradicate the glioblastomas, and at the higher dose tested, it proved detrimental due to the excess swelling in the absence of concomitant chemotherapy. Further research regarding dose effectiveness and safety margins will be required for translation of these results to the human clinical setting. The results further suggest that Talpha1 had little or no direct cytotoxic effects on malignant neoplastic cells, and that the additional tumor cell killing observed with Talpha1+BCNU treatment was mediated by indirect actions of Talpha1. Since there were no apparent differences in the densities of lympho-mononuclear cells infiltrating the tumors, it is unlikely that the Talpha1-mediated effects were due to recruitment of inflammatory cells. Therefore, we elected not to pursue extensive analysis of the inflammatory cell infiltrates using the in vivo model. Moreover, we anticipated that such data would be skewed by differences in tissue swelling and tumor necrosis associated with the different treatments. Instead, in vitro experiments were conducted to clarify the potential mechanism by which Talpha1 enhanced the cytotoxic actions of BCNU against glioblastoma cells. 

The in vitro studies demonstrated that Talpha1 had no significant direct cytotoxic effects on the glioblastoma cells, consistent with the in vivo findings. Similar results were obtained with respect to other cell types including SH-Sy5y neuroblastoma cells, 293 kidney cells, C6 glioma cells, and postmitotic cortical neurons. However, it was effective in reducing tumor volume when administered with BCNU via mechanisms that did not involve increased inflammation or impairments in cell motility/invasiveness. Therefore, we hypothesized that as an adjuvant agent, Talpha1 may help mediate tumor cell killing by enhancing activation of proapoptosis or oxidative stress pathways. To extend these investigations, we evaluated whether Talpha1 adversely affected cell function and perhaps rendered the cells more sensitive to injurious agents including oxidants. This was done by examining the expression levels of proapoptosis and prosurvival genes, as well as growth and housekeeping genes. Those studies revealed that Talpha1 treatment for as little as 24 hours resulted in significantly increase levels of proapoptosis genes in 9L glioblastoma cells. Similar results were obtained with C6 glioma cells; whereas in SH-Sy5y neuroblastoma and 293 kidney cells, Talpha1 treatment inhibited prosurvival mechanisms rather than stimulate proapoptosis genes. These findings in aggregate suggest that, although Talpha1 has no direct cytotoxic effects, it may render neoplastic cells more sensitive to cytotoxic agents by increasing the expression of proapoptosis genes or reducing expression of survival genes. To test this hypothesis, we determined if Talpha1-treated cells were more sensitive to oxidative stress or BCNU-mediated killing. The studies showed that after 24 hours of Talpha1 treatment, sublethal concentrations of H_2_O_2_ or BCNU, respectively, killed 25% or 40% of the 9L cells. Therefore, at least some of the effects of Talpha1 were mediated by its actions on the neoplastic cells rather than by immune modulation and recruitment/activation of T cells, NK cells, and macrophages. Although the mechanism of these effects is not known, the findings in previous reports suggest that Talpha1 can have direct effects on the biological function of nonimmune cells [25, 56–59], including inhibition of neoplastic neural and glial cell proliferation [24, 25], enhancement of the proapoptosis actions of compounds such as retinoic acid [56], and promoting the conversion of Cytochrome C from an antioxidant to pro-oxidant form of the molecule [57]. The latter may represent the mechanism by which Talpha1 increased the proapoptosis gene expression in the 9L glioblastoma cells.

The immune-modulating properties of Talpha1 and related molecules have been well established. Its major effects are to increase proinflammatory cytokine production and lymphocyte proliferation. Activated T lymphocytes kill target cells through FasL-FasR interactions and by activating the perforin-granzyme system. The finding of increased FasL and FasR expression in Talpha1-treated 9L glioblastoma cells suggests that activated T cells could effectively kill these target cells though FasL/FasR interactions. Utilizing a novel in vitro assay constructed with SLO (permeabilizing agent) and recombinant Granzyme B instead of activated T cells, we demonstrated that Talpha1-treated 9L glioblastoma cells were rapidly killed by exposure to SLO and Granzyme B. These findings suggest that Talpha1 may effectively promote immune-mediated killing of glioblastoma cells in several ways: (1) increasing basal levels of proapoptosis gene expression rendering the cells more sensitive to oxidative stress and cytotoxic/chemotherapeutic agents; (2) increasing levels of FasR which could interact with FasL on activated T cells or NK cells and lead to increased apoptosis; (3) enhancing perforin-granzyme-mediated killing of target tumor cells. In addition, the results strongly indicate that Talpha1 treatment of glioblastomas is effective when used in combination with BCNU, but not as a stand-alone agent. The results provide substantial evidence that Talpha1 administered alone may be detrimental due to increased swelling and inflammation with minimal tumor cell eradication. Therefore, the most suitable role for Talpha1 in the treatment of glioblastomas is as an adjuvant agent for boosting the host immune response and eradicating residual tumor cells that survive conventional chemotherapy.

In conclusion, the work described herein demonstrates that Talpha1 exhibits antiglioblastoma effects that are mediated through several channels including: (1) modulation of proapoptosis/survival genes leading to increased tumor cell sensitivity to oxidative stress or cytotoxic/chemotherapeutic agents; (2) promoting FasR-FasL-mediated immune cell killing cascades; (3) increasing target cell sensitivity to perforin-granzyme-mediated immune cell killing. The finding that the therapeutic effects of Talpha1 with respect to its antiglioblastoma properties were dependent upon the concomitant administration of BCNU emphasizes that Talpha1 would be best suited as an adjuvant immune modulator rather than a definitive antineoplastic agent. It is also likely that when combined with other chemotherapeutic compounds, Talpha1 could have similar positive effects in helping to reduce tumor burden, progression, and recurrences at significantly greater rates than currently observed with conventional chemotherapy.

## References

[1] Burton EC, Prados MD (2000). Malignant gliomas. *Current Treatment Options in Oncology*.

[2] Gal H, Makovitzki A, Amariglio N, Rechavi G, Ram Z, Givol D (2007). A rapid assay for drug sensitivity of glioblastoma stem cells. *Biochemical and Biophysical Research Communications*.

[3] Kleihues P, Louis DN, Scheithauer BW (2002). The WHO classification of tumors of the nervous system. *Journal of Neuropathology and Experimental Neurology*.

[4] Macdonald DR (2001). Temozolomide for recurrent high-grade glioma. *Seminars in Oncology*.

[5] Prados MD, Levin V (2000). Biology and treatment of malignant glioma. *Seminars in Oncology*.

[6] Nieder C, Grosu AL, Molls M (2000). A comparison of treatment results for recurrent malignant gliomas. *Cancer Treatment Reviews*.

[7] Napolitano M, Keime-Guibert F, Monjour A (1999). Treatment of supratentorial glioblastoma multiforme with radiotherapy and a combination of BCNU and tamoxifen: a phase II study. *Journal of Neuro-Oncology*.

[8] Dazzi C, Cariello A, Giannini M (2000). A sequential chemo-radiotherapeutic treatment for patients with malignant gliomas: a phase II pilot study. *Anticancer Research*.

[9] Back MF, Ang ELL, Ng W-H (2007). Improved median survival for glioblastoma multiforme following introduction of adjuvant temozolomide chemotherapy. *Annals of the Academy of Medicine Singapore*.

[10] Dix AR, Brooks WH, Roszman TL, Morford LA (1999). Immune defects observed in patients with primary malignant brain tumors. *Journal of Neuroimmunology*.

[11] Roth W, Weller M (1999). Chemotherapy and immunotherapy of malignant glioma: molecular mechanisms and clinical perspectives. *Cellular and Molecular Life Sciences*.

[12] Sablotzki A, Ebel H, Muhling J (2000). Dysregulation of immune response following neurosurgical operations. *Acta Anaesthesiologica Scandinavica*.

[13] Billich A (2002). Thymosin *α*1. SciClone Pharmaceuticals. *Current Opinion in Investigational Drugs*.

[14] Bodey B (2001). Thymic hormones in cancer diagnostics and treatment. *Expert Opinion on Biological Therapy*.

[15] Bodey B, Bodey B, Siegel SE, Kaiser HE (2000). Review of thymic hormones in cancer diagnosis and treatment. *International Journal of Immunopharmacology*.

[16] Andreone P, Cursaro C, Gramenzi A (2001). In vitro effect of thymosin-*α*1 and interferon-*α* on Th1 and Th2 cytokine synthesis in patients with chronic hepatitis C. *Journal of Viral Hepatitis*.

[17] Attia WY, Badamchian M, Goldstein AL, Spangelo BL (1993). Thymosin stimulates interleukin-6 production from rat spleen cells in vitro. *Immunopharmacology*.

[18] Baxevanis CN, Frillingos S, Seferiadis K (1990). Enhancement of human T lymphocyte function by prothymosin *α*: increased production of interleukin-2 and expression of interleukin-2 receptors in normal human peripheral blood T lymphocytes. *Immunopharmacology and Immunotoxicology*.

[19] Baxevanis CN, Gritzapis AD, Dedoussis GVZ, Papadopoulos NG, Tsolas O, Papamichail M (1994). Induction of lymphokine-activated killer activity in mice by prothymosin *α*. *Cancer Immunology Immunotherapy*.

[20] Beuth J, Schierholz JM, Mayer G (2000). Thymosin *α*1 application augments immune response and down-regulates tumor weight and organ colonization in 
BALB/c-mice. *Cancer Letters*.

[21] Cordero OJ, Sarandeses CS, Lopez JL, Nogueira M (1992). Prothymosin *α* enhances human natural killer cell cytotoxicity: role in mediating signals for NK activity. *Lymphokine and Cytokine Research*.

[22] Garbin F, Eckert K, Immenschuh P, Kreuser ED, Maurer HR (1997). Prothymosin *α*1 effects, in vitro, on the antitumor activity and cytokine production of blood monocytes from colorectal tumor 
patients. *International Journal of Immunopharmacology*.

[23] Knutsen AP, Freeman JJ, Mueller KR, Roodman ST, Bouhasin JD (1999). Thymosin-*α*1 stimulates maturation of CD34^+^ stem cells into CD3^+^4^+^ cells in an in vitro thymic epithelia organ coculture model. *International Journal of Immunopharmacology*.

[24] Spangelo BL, Farrimond DD, Pompilius M, Bowman K-L (2000). Interleukin-1*β* and thymic peptide regulation of pituitary and glial cell cytokine expression and cellular proliferation. *Annals of the New York Academy of Sciences*.

[25] Spangelo BL, Farrimond DD, Thapa M (1998). Thymosin fraction 5 inhibits the proliferation of the rat neuroendocrine MMQ pituitary adenoma and C6 glioma cell lines in vitro. *Endocrinology*.

[26] Tijerina M, Gorospe WC, Bowman K-L, Badamchian M, Goldstein AL, Spangelo BL (1997). A novel thymosin peptide stimulates interleukin-6 release from rat C6 glioma cells in vitro. *NeuroImmunoModulation*.

[27] Chan HL-Y, Tang J-L, Tam W, Sung JJ-Y (2001). The efficacy of thymosin in the treatment of chronic hepatitis B virus infection: a meta-analysis. *Alimentary Pharmacology and Therapeutics*.

[28] Chien R-N, Lin C-Y, Yeh C-T, Liaw Y-F (2006). Hepatitis B virus genotype B is associated with better response to thymosin *α*1 therapy than genotype C. *Journal of Viral Hepatitis*.

[29] Ideo G, Bellobuono A (2002). New therapies for the treatment of chronic hepatitis C. *Current Pharmaceutical Design*.

[30] Lau GKK, Nanji A, Hou J (2002). Thymosin-*α*1 and famciclovir combination therapy activates T-cell response in patients with chronic hepatitis B virus infection in immune-tolerant phase. *Journal of Viral Hepatitis*.

[31] Saruc M, Yuceyar H, Kucukmetin N, Demir MA, Kandiloglu AR (2002). Combination thymosin-*α*1 and interferon-*α*2b in the treatment of anti-HBe-positive chronic hepatitis B in Turkey. *Hepato-Gastroenterology*.

[32] Sherman KE, Jones CC, Goldstein AL, Naylor PH (1991). Low thymosin *α*-1 concentrations in patients chronically infected with the hepatitis B virus. *Viral Immunology*.

[33] Sherman KE, Sjogren M, Creager RL (1998). Combination therapy with thymosin *α*1 and interferon for the treatment of chronic hepatitis C infection: a randomized, placebo-controlled 
double-blind trial. *Hepatology*.

[34] Gramenzi A, Cursaro C, Andreone P, Bernardi M (1998). Thymalfasin: clinical pharmacology and antiviral applications. *BioDrugs*.

[35] Garaci E, Pica F, Rasi G, Favalli C (2000). Thymosin *α* 1 in the treatment of cancer: from basic research to clinical application. *International Journal of Immunopharmacology*.

[36] Ji SM, Li LS, Sun QQ, Chen JS, Sha GZ, Liu ZH (2007). Immunoregulation of thymosin *α* 1 treatment of cytomegalovirus infection accompanied with acute respiratory distress syndrome after renal transplantation. *Transplantation Proceedings*.

[37] Wang B, He F, Lin Y, Huang M, Zhou SF (2007). Effect of recombinant human thymosin-*α*1, an immuno-modulating peptide with 28 amino acids, on the activity of cytochrome P450s. *Drug Metabolism Letters*.

[38] de La Monte SM, Ganju N, Wands JR (1999). Microtiter immunocytochemical ELISA assay. *BioTechniques*.

[39] de la Monte SM, Neely TR, Cannon J, Wands JR (2001). Ethanol impairs insulin-stimulated mitochondrial function in cerebellar granule neurons. *Cellular and Molecular Life Sciences*.

[40] Ochel H-J (2005). Correlation between crystal violet dissolution assay and manual colony counting on the in vitro effects of Hsp90-inhibitors. *Journal of Experimental Therapeutics and Oncology*.

[41] de la Monte SM, Lahousse SA, Carter J, Wands JR (2002). ATP luminescence-based motility-invasion assay. *BioTechniques*.

[42] Davis JE, Sutton VR, Smyth MJ, Trapani JA (2000). Dependence of granzyme B-mediated cell death on a pathway regulated by Bcl-2 or its viral homology, BHRF1. *Cell Death and Differentiation*.

[43] Hayashida M, Kawano H, Nakano T, Shiraki K, Suzuki A (2000). Cell death induction by CTL: perforin/granzyme B system dominantly acts for cell death induction in human hepatocellular carcinoma cells. *Proceedings of the Society for Experimental Biology and Medicine*.

[44] Kontani K, Sawai S, Hanaoka J, Tezuka N, Inoue S, Fujino S (2001). Involvement of granzyme B and perforin in suppressing nodal metastasis of cancer cells in breast and lung cancers. *European Journal of Surgical Oncology*.

[45] Pipkin ME, Lieberman J (2007). Delivering the kiss of death: progress on understanding how perforin works. *Current Opinion in Immunology*.

[46] Simon MM, Waring P, Lobigs M (2000). Cytotoxic T cells specifically induce Fas on target cells, thereby facilitating exocytosis-independent induction of apoptosis. *Journal of Immunology*.

[47] Vermijlen D, Froelich CJ, Luo D, Suarez-Huerta N, Robaye B, Wisse E (2001). Perforin and granzyme B induce apoptosis in FasL-resistant colon carcinoma cells. *Cancer Immunology, Immunotherapy*.

[48] Vujanovic NL (2001). Role of TNF family ligands in antitumor activity of natural killer cells. *International Reviews of Immunology*.

[49] Sepe PS, Lahousse SA, Gemelli B (2002). Role of the aspartyl-asparaginyl-beta-hydroxylase gene in neuroblastoma cell motility. *Laboratory Investigation*.

[50] Baxevanis CN, Sfagos C, Anastasopoulos E, Reclos GJ, Papamichail M (1990). Prothymosin-*α* enhances HLA-DR antigen expression on monocytes from patients with multiple sclerosis. *Journal of Neuroimmunology*.

[51] Baxevanis CN, Reclos GJ, Economou M (1988). Mechanism of action of prothymosin *α* in the human autologous mixed lymphocyte response. *Immunopharmacology and Immunotoxicology*.

[52] Baxevanis CN, Reclos GJ, Panneerselvam C, Papamichail M (1988). Enhancement of human T lymphocyte functions by prothymosin *α*. I. Augmentation of mixed lymphocyte culture reactions and soluble protein-induced proliferative responses. *Immunopharmacology*.

[53] Di Francesco P, Pica F, Marini S, Favalli C, Garaci E (1992). Thymosin *α* one restores murine T-cell-mediated responses inhibited by in vivo cocaine administration. *International Journal of Immunopharmacology*.

[54] Hsia J, Sarin N, Oliver JH, Goldstein AL (1989). Aspirin and thymosin increase interleukin-2 and interferon-*γ* production by human peripheral blood lymphocytes. *Immunopharmacology*.

[55] Sztein MB, Serrate SA (1989). Characterization of the immunoregulatory properties of thymosin *α* 1 on interleukin-2 production and interleukin-2 receptor expression in normal human lymphocytes. *International Journal of Immunopharmacology*.

[56] Hall AK (1996). Liarozole amplifies retinoid-induced apoptosis in human prostate cancer cells. *Anti-Cancer Drugs*.

[57] Markova OV, Evstafieva AG, Mansurova SE (2003). Cytochrome c is transformed from anti- to pro-oxidant when interacting with truncated oncoprotein prothymosin *α*. *Biochimica et Biophysica Acta*.

[58] Naz RK, Kaplan P, Badamchian M, Goldstein AL (1995). Effects of synthetic thymosin-*α*1 and its analogs on fertilizability of human sperm: search for a biologically active, stable epitope. *Archives of Andrology*.

[59] Naz RK, Menge AC, Sacco A (1995). Treatment with thymosin *α*-1 increases fertilizing capacity of sperm of infertile men: a multicenter trial. *Archives of Andrology*.

